# Calcium-activated potassium channels in ischemia reperfusion: a brief update

**DOI:** 10.3389/fphys.2014.00381

**Published:** 2014-10-06

**Authors:** Jean-Yves Tano, Maik Gollasch

**Affiliations:** ^1^Experimental and Clinical Research Center, Charité University Medicine - Max Delbrück Center (MDC) for Molecular MedicineBerlin, Germany; ^2^Nephrology/Intensive Care Section, Charité University MedicineBerlin, Germany

**Keywords:** ischemia-reperfusion, K_Ca_ channels, potassium channels, cardiovascular

## Abstract

Ischemia and reperfusion (IR) injury constitutes one of the major causes of cardiovascular morbidity and mortality. The discovery of new therapies to block/mediate the effects of IR is therefore an important goal in the biomedical sciences. Dysfunction associated with IR involves modification of calcium-activated potassium channels (K_Ca_) through different mechanisms, which are still under study. Respectively, the K_Ca_ family, major contributors to plasma membrane calcium influx in cells and essential players in the regulation of the vascular tone are interesting candidates. This family is divided into two groups including the large conductance (BK_Ca_) and the small/intermediate conductance (SK_Ca_/IK_Ca_) K^+^ channels. In the heart and brain, these channels have been described to offer protection against IR injury. BK_Ca_ and SK_Ca_ channels deserve special attention since new data demonstrate that these channels are also expressed in mitochondria. More studies are however needed to fully determine their potential use as therapeutic targets.

## Introduction

The proper function of the vasculature requires an intricate balance between plasma membrane ion channels embedded in the endothelium and smooth muscle cells (Luksha et al., 2009). In this regard, the calcium-activated potassium channels (K_Ca_) exert a great influence in this process (Brayden and Nelson, 1992; Félétou, 2009). These potassium channels possess high sensitivity to intracellular calcium as well as to changes in membrane voltage (Yang et al., 2012). Vascular dysfunction, which is a characteristic trait of several pathophysiological problems such as ischemia-reperfusion (IR) injury, is usually associated with a breakdown of mechanisms in the endothelium or smooth muscle cells. Many of these mechanisms involve the contribution of ion channels including the K_Ca_. Due to their importance in the regulation of the vascular tone, the plasma membrane K_Ca_ channels have been under scrutiny to resolve vascular dysfunction. Consequently, their role in IR injury has been uncovered with the use of pharmacological tools and more recently with animal models. Our objective in this mini-review is to highlight the observed beneficial effect of K_Ca_ channels under IR conditions.

## Structure and function of K_Ca_ channels

**Figure 1:**
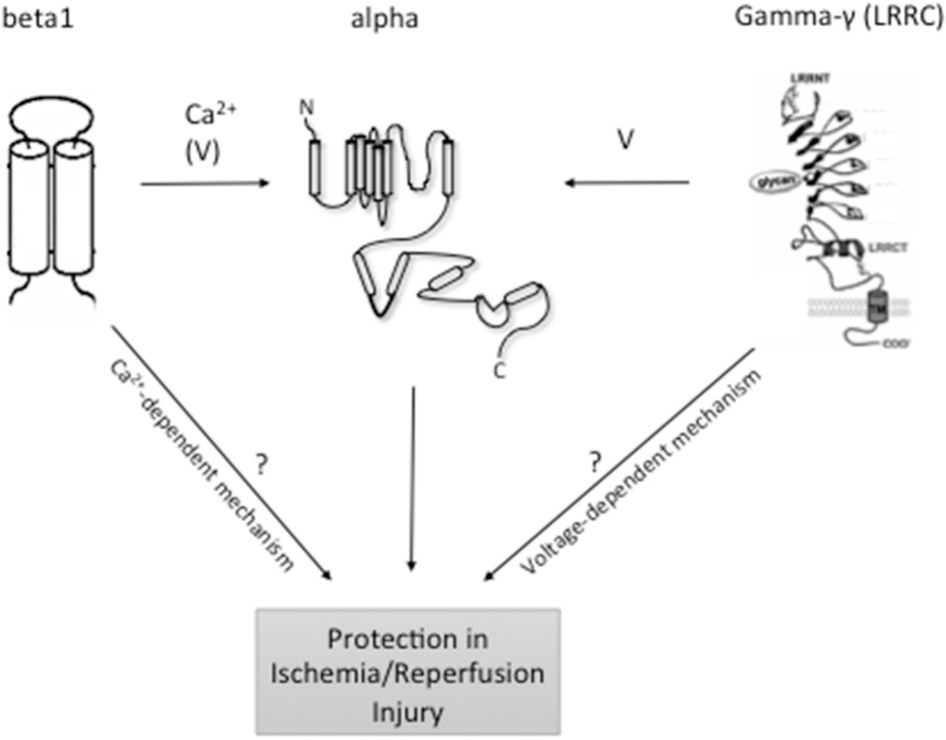
**Topology of BK_Ca_ and modulatory subunits**. At the plasma membrane, the N-terminus of BK_Ca_ α-subunits is extracellular, and the C-terminus is intracellular. Orientation in organelles is unknown. S0–S4 transmembrane domains are involved in voltage sensing. The S5–S6 linker lines the K+ selective pore. Four α-subunits are needed to form a functional channel. β 1–β 4 subunits have two transmembrane domains. N- and C-termini are facing the same side of the membrane. γ (LRRC)-subunits have a single transmembrane domain. N- and C-termini face opposite sides of the membrane. ß subunits have a major impact on the intracellular Ca^2+^ sensitivity of the channels, whereas γ subunits have major effects on BK_Ca_ channel voltage sensitivity to different degrees. Following reperfusion, an exacerbated accumulation of [Ca^2+^]_i_ mainly in the mitochondria, along with a significant increase in ROS and inflammation may result in cellular death. At least, mitoBK_Ca_ channels play a protective role against IR injury through thus far unclear mechanisms. LRRC exhibit tissue-specific expression although which individual cell types express LRRC proteins is unclear. Organ- and organelle-specific deletion of BK_Ca_ LRRC and ß subunits may clarify the role of [Ca^2+^]_i_ accumulation vs. membrane potential in the protective effects of BK_Ca_ channels in IR.

On the basis of structure, the K_Ca_ family of potassium channels comprises two groups (Wei et al., 2005). Due to sequence similarity in the pore region and in the C-terminal bound calmodulin Ca^2+^ sensing domain, the small-conductance (SK_Ca_1, 2, 3) and intermediate conductance (IK_Ca_1) belong to the same subgroup (Wei et al., 2005). The large-conductance BK_Ca_, Slo3, Slack, and Slick are also grouped together although Slo3, Slack, and Slick are insensitive to internal calcium (Wei et al., 2005) (see Table 1: for simplicity, only the Ca^2+^ activated potassium channels are shown). In contrast to the other members of the family, the BK_Ca_ channels are unique in that they are not only calcium but also markedly voltage sensitive and that calcium binds directly at a specific domain within the protein structure (Wei et al., 1994; Schreiber and Salkoff, 1997). BK_Ca_ channels can be in complex with several modulatory subunits (Figure 1) that greatly modify the channel kinetics and voltage/Ca^2+^ sensitivities: β 1–β 4 have two transmembrane domains, while leucine-rich repeat-containing proteins LRRC26, LRRC38, LRRC52, and LRRC55 are single pass membrane proteins with LRRC26 being the most potent activator producing a negative shift of approximately 140 mV of the voltage dependence of activation (Yan and Aldrich, 2010, 2012; Singh et al., 2012). LRRC26 is a functional BK Channel auxiliary γ subunit in arterial smooth muscle (Evanson et al., 2014). SK_Ca_ and IK_Ca_ channels, however, are very sensitive to changes in [Ca^2+^]_i_(submicromolar), whose activation of the channels depends on the binding to a constitutively attached calmodulin (Burnham et al., 2002; Bychkov et al., 2002). SK_Ca_ and IK_Ca_ are expressed predominantly in the endothelial cells whereas BK_Ca_ can be found in greater numbers in the smooth muscle cells (Yang et al., 2012). In the vasculature, these channels contribute predominantly in the regulation of the vascular tone.

**Table 1:** **Nomenclature of the calcium-activated potassium channels and their described participation in IR injury**.

**IUPHAR Name**	**Common name**	**HGNC**	**Role in IR injury**
K_Ca_1.1	Slo, Slo1, BK	*KCNMA1*	Heart: Protection
			Brain: Protection
K_Ca_2.1	SK_Ca_, SK_Ca_2	*KCNN1*	Heart: Protection
K_Ca_2.2		*KCNN2*	Brain: Protection
K_Ca_2.3		*KCNN3*	
K_Ca_3.1	IK_*Ca*,_ IK_Ca_1	*KCNN4*	Heart: Protection
			Brain: Protection

*All of the channels seem to provide protection against injury in the heart and the brain whether administered pre- or post-ischemia. Abbreviations: IUPHAR, International Union of Pharmacology; HGNC, HUGO Gene Nomenclature Committee; IR, Ischemia-Reperfusion*.

SK_Ca_ and IK_Ca_ in the endothelium facilitate the endothelial-derived hyperpolarizing factor mediated relaxation (EDHF) and more recently were found to be important for nitric oxide release (Doughty et al., 1999; McNeish et al., 2006; Stankevicius et al., 2006; Absi et al., 2007; Brähler et al., 2009). At least in mice, the EDHF response is caused by hydrogen peroxide, but not by cytochrome P450 eicosanoids (Hercule et al., 2009). In effect, following an increase in [Ca^2+^]_i_ in endothelial cells, SK_Ca_, and IK_Ca_ channels open, causing membrane hyperpolarization. Local calcium (Ca^2+^) signals (“sparklets”) generated through cooperative opening of individual TRPV4 channels within a four-channel cluster can open plasma membrane IK_Ca_ and SK_Ca_ channels to cause vasodilation (Sonkusare et al., 2012). The hyperpolarization in turn leads to the electrical coupling of the endothelium and smooth muscle cells through myoendothelial gap junctions and vasorelaxation (Félétou, 2009). In parallel, opening of these channels can cause activation of the inward rectifier Kir2.1 channels and/or the Na^+^/K^+^ ATPase on the smooth muscle cells, another important mechanism in the EDHF-mediated relaxation (Edwards et al., 1998). The coupling of the SK_Ca_ and IK_Ca_ channels activation to NO release is currently under study and involves several different mechanisms discussed extensively in Dalsgaard et al. (2010).

In arterial smooth muscle cells, BK_Ca_ channels are involved in regulation of the vascular tone primarily through hyperpolarization and limitation of calcium influx through Ca_v_1.2 L-type Ca^2+^ channels (Brayden and Nelson, 1992; Sausbier et al., 2005; Yang et al., 2012). Calcium sparks generated by opening of ryanodine receptors (RyR) in the sarcoplasmic reticulum serve as local elementary Ca^2+^ signals to open plasma membrane BK_Ca_ channels to induce membrane hyperpolarization and relaxation (Nelson et al., 1995; Gollasch et al., 1998; Essin et al., 2007), including in human vessels (Fürstenau et al., 2000). The accessory beta1 subunit of the BK_Ca_ channel plays an important role in calcium spark/BK channel coupling (Brenner et al., 2000; Plüger et al., 2000). Calcium sparks are possibly generated by opening of RyR2 (Essin and Gollasch, 2009; Vaithianathan et al., 2010), but not by RyR3 (Löhn et al., 2001). In addition, BK_Ca_ channels can contribute to endothelium-dependent vasorelaxation through activation by NO and EDHF (Bolotina et al., 1994; Weston et al., 2005; Hou et al., 2009). Interestingly, new studies have demonstrated the activation of BK channels by other gasotransmitters, notably carbon monoxide (CO) and hydrogen sulfide (H_2_S) (Dong et al., 2007; Chai et al., 2014) although see Telezhkin et al. (2010).

In view of their prominent role in the regulation of the vascular tone, the likelihood of involvement of these channels in IR—a condition where mechanisms underlying vasorelaxation are compromised and where gasotransmitters have been shown to play a protective role—is very high (Murphy and Steenbergen, 2008; Luksha et al., 2009; Dalsgaard et al., 2010; Eltzschig and Eckle, 2011). Recent studies have better defined the role of BK_Ca_ in IR, however the picture concerning SK_Ca_ and IK_Ca_ remains still cloudy.

## SK_Ca_ and IK_Ca_ in ischemia-reperfusion

A primary mechanism involved in IR injury is the exacerbation of intracellular calcium, which causes damages in the tissue (discussed in more detail in Eltzschig and Eckle, (2011), Tano and Gollasch, (2014). Limited studies have looked at the role of SK_Ca_ and IK_Ca_ in IR injury. Yang et al. recently demonstrated a decrease in endothelial IK_Ca_ and SK_Ca_ currents as well as IK_Ca_ protein content, associated with a decreased EDHF-mediated relaxation following 60 min ischemia and 30 min reoxygenation in pig arteries (Yang et al., 2011). This study suggests that these channels are important in the protection of the endothelium against IR injury. A more recent and rigorous study looking at isolated guinea pig hearts also found protection against IR injury through SK_Ca_ channels (Stowe et al., 2006). In this study, DCEBIO, an SK_Ca_, and IK_Ca_ channel activator, caused a 2-fold increase in left ventricular pressure as well as a 2.5 fold decrease in infarct size when administered for 10 min, 20 min before IR. This effect is, however, blocked by NS8593, an SK_Ca_ blocker, suggesting that these channels are responsible for the protection. Interestingly and most importantly, the authors isolate and purify novel mSK_Ca_ channels from the inner mitochondrial membrane of cardiac cell and suggest that DCEBIO mediates its cardioprotection through these channels (Stowe et al., 2006), by improving mitochondrial bioenergetics (Stowe et al., 2013).

In the brain, a few studies have also demonstrated a protective role of SK_Ca_ and a more ambiguous role for IK_Ca_. In mice undergoing cardiac arrest/cardiopulmonary resuscitation (CA/CPR) and global cerebral ischemia, SK_Ca_2 channels are responsible for the protection of the CA1 neurons against ischemic injury (Allen et al., 2011). Similarly to the study in the heart, pre-stimulation of SK_Ca_2 with 1-EBIO diminished significantly the adverse effects of CA/CPR, an effect, which could be reversed with administration of apamin (a specific SK_Ca_ blocker). In addition, SK_Ca_2 electrophysiological activity was reduced during CA/CPR in association with an increased synaptic SK_Ca_2 channels internalization. Interestingly, post-treatment with 1-EBIO was able to also blunt the effects of CA/CPR (Allen et al., 2011). In the parenchymal arterioles, both SK_Ca_ and IK_Ca_ were shown to play a protective role on the basal tone and pressure reactivity following IR (Cipolla et al., 2009). Blockade of these channels in the parenchymal arterioles induced a significant increase in the basal tone, which was preserved following IR injury when compared to control animals. Furthermore, the authors suggest that EDHF act as a substitute for NO in the parenchymal arterioles due to the fact that NO responsiveness is significantly decreased after IR (Cipolla et al., 2009). Finally, a recent study demonstrated that inhibition of IK_Ca_ with the blocker TRAM-34 reduces infarct size and other neurological deficits in rats when administered as soon as 12 h after middle cerebral artery occlusion (Chen et al., 2011). The mechanism suggested for the protective actions of this drug is through the reduced activation of microglial cells, which is more noticeable with a higher dose (40 mg/Kg) of TRAM-34 (Chen et al., 2011).

Since the studies described in this review represent the only few published on this topic, one can see that much more work is required to properly decipher the role of these important channels in IR injury. It is especially difficult to understand the role of these channels since most of these studies take very different pharmacological approaches, notably pre-, and post-administration of inhibitors or blockers in conjunction with IR. Moreover, the use of available knockout mouse models of these channels would bring the scientific community closer to this goal. The prominent trend, however, seems to be a protective effect of these channels in the heart and the brain (see Table 1), which is also evident for BK_Ca_ channels.

## BK_Ca_ in ischemia-reperfusion

The combination of pharmacological tools and knockout mouse models has suggested a protective role of BK_Ca_ against IR injury. The use of pharmacological activators such as NS1619 and NS11021 suggested BK_Ca_ channels as cardioprotective following IR (Shintani et al., 2004; Shi et al., 2007; Bentzen et al., 2009). This notion was recently confirmed with the use of the *Kcnma*1 knockout mouse where the cardioprotective effects of these channels were lost (Wojtovich et al., 2013). Furthermore, Woodman et al. determined the effects of tetraethylammonium (TEA, 1 mM—a potent blocking concentration for BK_Ca_ channels (see Nelson, 1993) to coronary arteries from dogs subjected to IR. TEA significantly shifted the concentration response curve of the ischemic vessels to acetylcholine to the right, though without decreasing the maximal relaxation (Chan and Woodman, 1999). The authors concluded that EDHF may be the factor responsible for activation of BK_Ca_ channels (Chan and Woodman, 1999). However, the data have to be interpreted with caution since a number of other K+ channels are sensitive to TEA, within this range of concentration, e.g., Kv1.1, Kv1.3, and Kv1.6 (Al-Sabi et al., 2013), KCNQ1, KCNQ2, KCNQ4, KCNQ2 + KCNQ3 (Hadley et al., 2000). In skeletal muscle arterioles from patients undergoing cardiopulmonary bypass, Feng et al. observed activation of the BK_Ca_ channels (Feng et al., 2009). In addition, treatment with iberiotoxin (a specific BK_Ca_ blocker) improved the myogenic tone significantly associated with a reduced microvessel internal diameter in these patients. The molecular mechanisms of the protective effects of BK_Ca_ channels in IR may involve direct effects of hypoxia on BK_Ca_ channel gating, without involvement of soluble intracellular components (Lewis et al., 2002). Sensitivity to hypoxia is conferred by a highly conserved motif within an alternatively spliced cysteine-rich insert, the stress-regulated exon (STREX), within the intracellular C-terminus of the channel (McCartney et al., 2005). Recent studies using *Kcnma*1 knockout mice suggest that activation of cardiomyocyte BK_Ca_ channels in mitochondria (mitoBK_Ca_) is one mechanism that protects the heart against IR injury (Singh et al., 2013; Tano and Gollasch, 2014). It is possible that sulfhydryl groups of the channel protein play a critical role in this process (Sitdikova et al., 2010; Liu et al., 2012).

The *Kcnma1* knockout mouse was also used to study BK_Ca_ channels in the brain. These channels offered protection and reduced infarct size in a middle cerebral artery occlusion model (Liao et al., 2010). Interestingly, Gu et al. found that unlike healthy brain cells, glioma mitoBK_Ca_ channels, but not plasma membrane BK channels are oxygen sensitive (Gu et al., 2014). These findings may explain why tumor cells are resistant to hypoxia. On the other hand, discovery of this mechanism of tumor tolerance may have important clinical implications for the development of novel therapies in oncology.

## Conclusion

The K_Ca_ play an essential function in the endothelium and arterial smooth muscle where they participate actively in the regulation of the myogenic tone. Disruption of this process as well as others such as NO formation in IR injury provides a reason to study a potential involvement of these channels in IR. Thus far, the consensus points toward a protective role of these channels against IR injury, although much more remains unknown, notably the mechanisms underlying this protection. The use of gene knockout mouse models, especially for the SK_Ca_, and IK_Ca_ would be of great help in answering these questions. Also, the very recent discovery of BK_Ca_ channel auxiliary γ subunits, such as LRRC26, LRRC38, LRRC52, and LRRC55 (Yan and Aldrich, 2010, 2012), may help to design experimental protocols to clarify the role of excess calcium vs. plasma/mito membrane potential in the protective BK_Ca_ function in IR injury (Figure 1). In this regard, targeting BK_Ca_ β subunits but not γ subunits is expected to affect IR injury if excess calcium plays a key role in this process. Future studies are necessary to address the composition of functional BK_Ca_ channels in the organs and organelles of interest (mitochondria) and to study their role in IR using genetically engineered BK_Ca_ subunit deficient animal models.

### Conflict of interest statement

The authors declare that the research was conducted in the absence of any commercial or financial relationships that could be construed as a potential conflict of interest.
